# Assessing the Effects of Opioids on Pathological Memory by a Computational Model

**DOI:** 10.32598/bcn.9.4.275

**Published:** 2018-07-01

**Authors:** Mehdi Borjkhani, Fariba Bahrami, Mahyar Janahmadi

**Affiliations:** 1. Motor Control and Computational Neuroscience Laboratory, School of Electrical & Computer Engineering, College of Engineering, University of Tehran, Tehran, Iran.; 2. Neuroscience Research Center, Shahid Beheshti University of Medical Sciences, Tehran, Iran.; 3. Department of Physiology, School of Medicine, Shahid Beheshti University of Medical Sciences, Tehran, Iran.

**Keywords:** Opioids, Memory of addiction, Synaptic plasticity, Long-Term Potentiation (LTP), Hippocampus

## Abstract

**Introduction:**

Opioids hijack learning and memory formation mechanisms of brain and induce a pathological memory in the hippocampus. This effect is mainly mediated by modifications in glutamatergic system. Speaking more precisely, Opioids presence in a synapse inhibits blockage of N-Methyl-D-Aspartate Receptor (NMDAR) by Mg^2+^, enhances conductance of NMDAR and thus, induces false Long-Term Potentiation (LTP).

**Methods:**

Based on experimental observations of different researchers, we developed a mathematical model for a pyramidal neuron of the hippocampus to study this false LTP. The model contains a spine of the pyramidal neuron with NMDAR, α-Amino-3-hydroxy-5-Methyl-4-isoxazole Propionic Acid Receptors (AMPARs), and Voltage-Gated Calcium Channels (VGCCs). The model also describes Calmodulin-dependent protein Kinase II (CaMKII) and AMPAR phosphorylation processes which are assumed to be the indicators of LTP induction in the synapse.

**Results:**

Simulation results indicate that the effect of inhibition of blockage of NMDARs by Mg^2+^ on the false LTP is not as crucial as the effect of NMDAR’s conductance modification by opioids. We also observed that activation of VGCCs has a dominant role in inducing pathological LTP.

**Conclusion:**

Our results confirm that preventing this pathological LTP is possible by three different mechanisms: 1. By decreasing NMDAR’s conductance; and 2. By attenuating VGCC’s mediated current; and 3. By enhancing glutamate clearance rate from the synapse.

## Highlights

- Opioids form pathological memories by induction of false LTP.
- Decrease in NMDAR conductance and attenuation of VGCC can prevent pathological LTP.
- Enhancing the glutamate clearance rate from the synapse can prevent false LTP.

## Plain Language Summary

Drug abuse creates pathological memories in different brain regions like the hippocampus. Information about the context of drug abuse is stored in hippocampus. In withdrawal times, the recall of pathological memories can trigger the relapse. Preventing the relapse is one of the challenges in the field of addiction research. Some researchers believe that if the pathological memory is not formed or weakened, the relapse will be undermined. To get a better understanding about the latter, we developed a computational model to simulate different cases. Our simulation results suggest that decreasing the conductance of glutamatergic receptors such as NMDARs or clearing the extra glutamates from the synapse, in other words, changing some of the synaptic characteristics can prevent formation of drug-related memories at the synaptic level. This will decrease the possibility of relapse during withdrawal period.

## 1. Introduction

Opioid consumption like other drugs can lead to behavioral changes (Contet, Kieffer, & Befort, 2004; Williams, Christie, & Manzoni, 2001). Some researchers believe that these changes are due to pathological memory formation which can be called memory of addiction (Dong & Nestler, 2014; Kelley, 2004; Nestler, 2001). Hippocampus seems to be one of the targets subject to the plastic changes due to drug abuse (Bao et al., 2007; Borjkhani, Mahdavi, & Bahrami, 2014; Capogna, Gähwiler, & Thompson, 1993; Caudle & Chavkin, 1990; Ga, 1980; Heidari et al., 2013; Hosseinmardi, Azimi, Fathollahi, Javan, & Naghdi, 2011; Hosseinmardi, Fathollahi, Naghdi, & Javan, 2009).

At the synaptic level, drug-induced changes might be due to Long-Term Potentiation (LTP)/Long-Term Depression (LTD) induction. Therefore, analyzing LTP/LTD induction in synapses under the presence of opioids can help researchers to unveil the formation mechanisms of this pathological memory by drugs (Kauer & Malenka, 2007; Lüscher & Malenka, 2011).

Opioids affect hippocampal neurons mainly by enhancing glutamate release in presynaptic neurons (Akaishi, Saito, Ito, Ishige, & Ikegaya, 2000; Williams et al., 2001) and increasing postsynaptic receptors activities (Kauer & Malenka, 2007; van Huijstee & Mansvelder, 2015; Williams et al., 2001). All the modifications caused by opioids result in LTP induction in affected synapses (Bao et al., 2007; Wolf, 2003). It has been shown that one of the causes of LTP induction is Calmodulin-dependent protein Kinase II (CaMKII) phosphorylation process (Lisman, Yasuda, & Raghavachari, 2012; Sanhueza & Lisman, 2013).

On the other hand, Ca^2+^ concentration is the main mediator in this process. Elevation of Ca^2+^ concentration in postsynaptic neuron triggers CaMKII phosphorylation process. Ca^2+^ can enter into the neuron through Voltage-Gated Calcium Channels (VGCCs), N-Methyl-D-Aspartate Receptor (NMDAR) and Ca^2+^ permeable α-Amino-3-hydroxy-5-methyl-4-isoxazole Propionic Acid Receptors (AMPARs). Thus, these channels and receptors contribute in CaMKII phosphorylation process and LTP induction.

It has been shown that blocking hippocampal VGCCs can prevent opioids dependency (Mishra, Barik, & Ray, 2017). Furthermore, opioid effects on NMDARs and AMPARs were verified in different research studies (Capogna et al., 1993; Chen & Huang, 1992; Chen & Marine, 1991; Ga, 1980; Kauer & Malenka, 2007; Nestler, 2013; Peters & De Vries, 2012; Williams et al., 2001). Most of the research and findings in this field are based on experimental observations.

However, computational models offer new tools and approaches for investigating neurophysiological basis of addiction memory formation process. In general, computational modeling of biological systems helps us to study consistency of different experimental findings and to predict the behavior of a given biological system under different constraints. That is why computational modeling has recently attracted the interests of many researchers in the field of neuroscience (Amiri, Bahrami, & Janahmadi, 2012; Amiri, Montaseri, & Bahrami, 2011; Li et al., 2016; Tewari & Majumdar, 2012b; Volman, Bazhenov, & Sejnowski, 2012).

Therefore, in this paper, using experimental findings we introduce a minimal computational model to assess opioid-induced LTP in CA1 region of the hippocampus. This model can also be used for analyzing the effects of other drugs by minor modifications. In the first part of the paper, opioids effect on hippocampal neurons will be discussed. The second part is dedicated to introduce mathematical modeling approach. The third part presents simulation results. Discussion and conclusion will be presented in the final part.

## 2. Methods

### 2.1 Neurophysiological background

According to many research studies, opioid-induced modifications in glutamatergic synapses are responsible for pathological memory formation related to the addiction (Harris, Wimmer, Byrne, & Aston-Jones, 2004; Herman et al., 2003; Mameli, Bellone, Brown, & Lüscher, 2011; van Huijstee & Mansvelder, 2015). Since Long-Term Potentiation (LTP)/Long-Term Depression (LTD) induction can be considered as signs of memory formation process at the synaptic level, one can also suggest that addiction memory formation might occur through the induction of LTP/LTD.

It has been shown that opioids induce LTP in hippocampal synapses by two primary mechanisms: 1. Enhancing glutamate release through disinhibitory mechanism (Capogna et al., 1993; Caudle & Chavkin, 1990; Cohen, Doze, & Madison, 1992; Rezai, Kieffer, Roux, & Massotte, 2013); and 2. By manipulating postsynaptic NMDARs current (Chen & Huang, 1992; Chen & Marine, 1991; Kow, Commons, Ogawa, & Pfaff, 2002; van Huijstee & Mansvelder, 2015). Opioids modify NMDARs current by increasing conduction of NMDARs and inhibiting Mg^2+^ from blocking NMDA receptors (Chen & Huang, 1992; Chen & Marine, 1991; Herman et al., 2003).

Consequently, these changes in synaptic transmission result in more calcium flux into the postsynaptic neuron (Kow et al., 2002; Przewlocki et al., 1999). Enhancement of postsynaptic calcium can activate CAMKII mechanisms (Herman et al., 2003; Trujillo, 2002). Phosphorylation of CaMKII leads to LTP induction and can be seen as molecular memory formation (Kauer & Malenka, 2007; Lisman et al., 2012; Sanhueza & Lisman, 2013). Furthermore, enhancement of postsynaptic calcium can lead to phosphorylation of AMPARs (Castellani, Quinlan, Bersani, Cooper, & Shouval, 2005; Castellani, Quinlan, Cooper, & Shouval, 2001; Kauer & Malenka, 2007).

**Figure 1.**
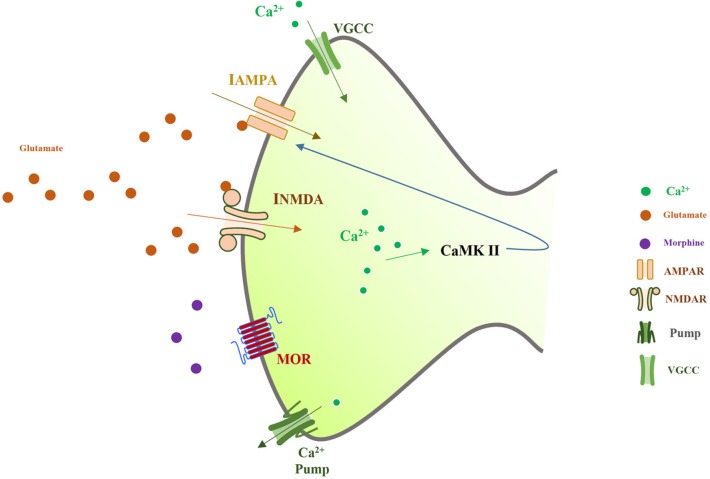
Spine of a CA^1^ pyramidal neuron which is modeled in this work As it is shown, synaptic glutamate activates AMPARs and NMDARs. By activation of these receptors, calcium enters into the neuron through VGCCs, NMDARs, and AMPARs. Activation of calcium pumps decreases calcium concentration in the neuron. Elevation of calcium may lead to activation of CaMKII mechanism which enhances AMPAR conductance.

Also, it has been shown that VGCCs are involved in opioids dependency (Bongianni, Carla, Moroni, & Pellegrini-Giampietro, 1986; Mishra et al., 2017). To investigate the mechanism of a false LTP induced by opioids, we developed a computational model of a postsynaptic neuron in CA1 region. In this model, we considered all important aspects mentioned from these neurophysiological studies. This model will be described in the next part (Figure 1).

### 2.2. Computational modeling approach

To analysis opioid-induced synaptic plasticity in CA1 region of the hippocampus, the spine of a CA1 pyramidal neuron is modeled by Equation (1):

(1) $$\;\tau_{\mathrm{post}}\;\frac{dV_{\mathrm{post}}}{dt}=-({\text{V}}_{\mathrm{post}}-{\text{V}}_{\mathrm{post}}^{\mathrm{rest}})+{\text{R}}_{m}\;I_{\mathrm{syn}}$$

This Equation denotes a minimal model to produce Excitatory Postsynaptic Potentials (EPSPs) based on Tsodyks and Markram’s passive membrane model (Tsodyks & Markram, 1997), where *τ_post_* shows time constant of neuron membrane, V*^rest^_post_* denotes neuron’s membrane potential at rest, and R*_m_* denotes actual resistance of spin. The synaptic current denoted by I*_syn_* can be described by:

(2) $$I_{\mathrm{syn}}\;=\;-({\text{I}}_{\mathrm{AMPA}}+{\text{I}}_{\mathrm{NMDA}})$$

Here, I*_AMPA_* and I*_NMDA_* are AMPAR and NMDAR currents, respectively. Model of AMPAR current is based on Destexhe’s model (Destexhe, Mainen, & Sejnowski, 1998):

(3) $$I_{\mathrm{AMPA}}\;=\;{\text{g}}_{\mathrm{AMPA}}m_{\mathrm{AMPA}}\left({\text{V}}_{\mathrm{post}}-{\text{V}}_{\mathrm{AMPA}}\right)$$

Where V*_AMPA_* refers to reversal potential of the receptor, V*_post_* is the membrane potential, and m*_AMPA_* denotes gating variable of AMPAR. Gating variable of AMPAR can be represented by the following Equation (Destexhe, Mainen, & Sejnowski, 1998):

(4) $$\frac{dm_{\mathrm{AMPA}}}{dt}=\alpha_{\mathrm{AMPA}}\;\;{\text{g}}_{\mathrm{pre}}(1-{\text{m}}_{\mathrm{AMPA}})-\beta_{\mathrm{AMPA}}m_{\mathrm{AMPA}}$$

Here opening and closing rate of the receptor are shown by α*_ampa_* and β*_ampa_*, respectively, g*_pre_* shows glutamate concentration, g*_AMPA_* described in Equation (3) denotes AMPAR’s channel conductance that can be enhanced due to CaMKII phosphorylation process by using the following Equation:

(5) $$g_{\mathrm{AMPA}}=g_{AMPA_{0}}(1+\frac{1}{1+e^{-(\text{CaMKII}-{\text{P}}_{\mathrm{half}})/{\text{k}}_{\mathrm{half}}}})$$

Where *g_AMPA0_*=0.4 *nS* is the initial value for AMPAR’s conductance, *P_half_*=40 *μM* and *k_half_*=0.4 *μM* are constants which enables AMPAR conductance to increase through CaMKII phosphorylation process. Model of the NMDAR current can described by the following Equation (Destexhe, Mainen, & Sejnowski, 1998; Moradi et al., 2013):

(6) $$I_{NMDA}=g_{\mathrm{NMDA}}m_{\mathrm{NMDA}}Mg({\text{V}}_{\mathrm{post}}-{\text{V}}_{\mathrm{NMDA}})$$

Where Mg shows Mg^2+^ blocking, represented by:

(7) Mg=1/(1+[Mg2+]0(k0+15.581+(0.1Op)1.2)−1exp⁡(−z(δ+0.11+(0.1Op)1.2)FVpostR−1T−1))

Moreover, NMDAR gating variable is described by:

(8) $$\frac{dm_{\mathrm{NMDA}}}{dt}=\alpha_{\mathrm{NMDA}}\;\;{\text{g}}_{pre}\;\left(1-m_{\mathrm{NMDA}}\right)-\beta_{\mathrm{NMDA}}\;m_{\mathrm{NMDA}}$$

Where V*_NMDA_* shows the reversal potential of NMDAR, opening and closing rate of receptor are shown by α*_NMDA_*and β*_NMDA_* respectively, Op refers to opioids concentration in the synapse, and g*_pre_* shows glutamate concentration. NMDA channels conductance (g*_NMDA_*) is represented by the following Equation:

(9) gNMDA=(gVI+0.151+(0.1Op)1.2)+gVD,

where g*_VI_* and g*_VD_* are voltage-independent and voltage-dependent conductance. The latter conductance is described by:

(10) $$\begin{array}{ll} \frac{dg_{VD}}{dt}= & ({\text{g}}_{VD,\infty }-{\text{g}}_{VD})/\tau_{g}\\ {\text{g}}_{VD,\infty }= & k({\text{V}}_{\mathrm{post}}-{\text{V}}_{0}) \end{array}$$

Here, g_*VD*,∞_ is the final value of g*_VD_, τ_g_* denotes time constant, g_*VD,*∞_, and V*_post_* has a linear relation with constant of K. Other parameters and their values have been listed in the Appendix.

Calcium can enter into the neuron through AMPARs, NMDARs, and VGCCs. Also, calcium pumps are responsible for transferring the calcium out of the neuron. So, calcium concentration inside the neuron can be represented by the following Equation (Tewari & Majumdar, 2012b:

(11) $$\begin{array}{l} \frac{dc_{\mathrm{post}}}{dt}=\frac{f({\text{c}}_{\mathrm{post}})}{1+\theta }\\ f(c_{\mathrm{post}})=-\frac{\left(\eta I_{\mathrm{AMPA}}+\gamma I_{\mathrm{NMDA}}+I_{R}\right)}{Z_{\mathrm{ca}}{\mathrm{FV}}_{\mathrm{spine}}}-S_{\mathrm{pump}}\\ \theta =\frac{b_{t}K_{\mathrm{endo}}}{{\left(K_{\mathrm{endo}}+c_{\mathrm{post}}\right)}^{2}} \end{array}$$

Here, C*_post_* shows postsynaptic calcium concentration, I*_R_* denotes voltage-gated calcium channels activation, and S*_pump_* refers to pumped calcium. Here, *η*=0.012 and *y*=0.06 demonstrate the amount of calcium that can enter into the neuron through AMPARs and NMDARs. Z_ca_=2 denotes calcium valence, F=96487C/*mo l* is the faraday’s constant, and V_*spine*_=0.9048 *μM*^3^ is the volume for dendrite spin. Furthermore, b_t_=200 *μM* shows total endogenous buffer concentration and K*_endo_*=10 *μM* denotes endogenous buffer calcium affinity. Pumped calcium is represented by the following Equation (Tewari & Majumdar, 2012b):

(12) $$S_{\mathrm{pump}}=k_{s}\left(c_{\mathrm{post}}-c_{\mathrm{post}}^{\mathrm{rest}}\right)$$

Here, K*_s_*=100/s is the maximum efflux rate of calcium pump, C*^rest^_post_*=100*nM* is the rest value for postsynaptic calcium concentration, and VGCCs activation is represented by the following Equation (Tewari & Majumdar, 2012b):

(13) $$I_{R}=g_{R}B(\text{N},{\text{P}}_{\mathrm{open}})({\text{V}}_{\mathrm{past}}-{\text{V}}_{R})$$,

where g*_R_*=15*ps* is the conductance of calcium channel, B(N, P*_open_*) denotes a random variable with a binomial distribution which shows the number of opened channels, and V*_R_*=27.4*mv* denotes the reversal potential.

Phosphorylation of CaMKII due to calcium concentration can be described by the following Equation (Zhabotinsky, 2000):

(14) $$\begin{array}{l} \frac{dP_{0}}{dt}=-v_{1}+v_{3}P_{1},\frac{dP_{1}}{dt}=v_{1}-v_{3}P_{1}-v_{2}P_{1}+2v_{3}P_{2},\\ \frac{dP_{2}}{dt}=v_{2}P_{1}-2v_{3}P_{2}-1.8v_{2}P_{2}+3v_{3}P_{3}\\ \frac{dP_{3}}{dt}=1.8v_{2}P_{2}-3v_{3}P_{3}-2.3v_{2}P_{3}+4v_{3}P_{4},\\ \frac{dP_{4}}{dt}=2.3v_{2}P_{3}-4v_{3}P_{4}-2.7v_{2}P_{4}+5v_{3}P_{5}\\ \frac{dP_{5}}{dt}=2.7v_{2}P_{4}-5v_{3}P_{5}-2.8v_{2}P_{5}+6v_{3}P_{6},\\ \frac{dP_{6}}{dt}=2.8v_{2}P_{5}-6v_{3}P_{6}-2.7v_{2}P_{6}+7v_{3}P_{7}\\ \frac{dP_{7}}{dt}=2.7v_{2}P_{6}-7v_{3}P_{7}-2.3v_{2}P_{7}+8v_{3}P_{8},\\ \frac{dP_{8}}{dt}=2.3v_{2}P_{7}-8v_{3}P_{8}-1.8v_{2}P_{8}+9v_{3}P_{9}\\ \frac{dP_{9}}{dt}=1.8v_{2}P_{8}-9v_{3}P_{9}-v_{2}P_{9}+10v_{3}P_{10},\\ \frac{dP_{10}}{dt}=v_{2}P_{9}-10v_{3}P_{10}\\ \frac{de_{p}}{dt}=-k_{3}Ie_{p}+k_{4}({\text{e}}_{p0}-{\text{e}}_{p}),\frac{dI}{dt}=-k_{3}Ie_{p}+k_{4}\\ ({\text{e}}_{p0}-{\text{e}}_{p})+{\text{v}}_{PKA}I_{0}-\frac{v_{CaN}{\left([Ca^{2+}]/K_{H2}\right)}^{3}I}{1+{\left([{\text{Ca}}^{2+}]{/}_{H2}\right)}^{3}} \end{array}$$,

where *P_i_* shows the concentration of the i-fold phosphorylated CaMKII, *e_p_* refers to the PP1 concentration which is not bounded to l1P and may demonstrate active protein phosphatase, the total concentration of PP1 is shown by *e*_*p*0_=0.1 *μM*, free l1P is denoted by *I*, and free l1 concentration is shown by *I*_0_=0.1 *μM*. Also *K*_3_=1/*μMs* and *K*_3_=10^−3^/*s* are association and dissociation rate constant of PP1-l1P complex, respectively. v*_caN_*=2/s refers to the rate of l1P dephosphorylation due to calcineurin (CaN), V*_PKA_*=0.45 μM/sis the phosphorylation rate of l1 due to the protein kinase A (PKA), and K*_H2_*=0.7 *μM* is the calcium activation Hill constant of CaN.

Phosphorylation (*V*_1_), auto-phosphorylation (*V*_2_), and dephosphorylation (*V*_3_) rates can be represented by the following equations:

(15) $$\begin{array}{l} v_{1}=\frac{10k_{1}{\left([{\text{Ca}}^{2+}]/{\text{K}}_{H1}\right)}^{8}P_{0}}{{\left(1+{\left([{\text{Ca}}^{2+}]/{\text{K}}_{H1}\right)}^{4}\right)}^{2}}\\ v_{2}=\frac{k_{1}{\left([{\text{Ca}}^{2+}]/{\text{K}}_{H1}\right)}^{4}}{1+{\left([{\text{Ca}}^{2+}]/{\text{K}}_{H1}\right)}^{4}}\\ v_{3}=\frac{k_{2}e_{p}}{K_{M}+\sum_{1}^{10}iP_{i}} \end{array}$$,

where *k*_1_*=*0.5/*s* is the l1 dependent regulation rate of PP1 and K*_H_*_1_=4 *μM* is the Hill constant of CaMKII for calcium activation. K_M_=20 *μM* and are K_2_=10*/s* the Michaelis and catalytic constants, respectively. Finally, phosphorylated CaMKII can be presented by:

(16) $$Ph.CaMKII=\sum_{i=1}^{10}P_{i}$$

In the next part, simulation results are presented.

## 3. Results

The mathematical modeling approach simulation results are presented in this section. Simulations were implemented in Matlab 2014a. To solve differential equations, the forward Euler method with constant step size of 0.05 ms is used. Synaptic glutamate is assumed as a periodic signal with a frequency of 5 Hz which each pulse has a duration of 4 ms. The amplitude of this signal is 0.2 mM and the simulation time is 10 s. In all Figures, 1 second of the simulation is depicted to clarify dynamics of the signals.

### 3.1. Normal and pathological conditions

One of the features of opioid-induced synaptic plasticity is the induction of LTP where normal LTP cannot occur (van Huijstee & Mansvelder, 2015). Therefore, stimulation signal (which is synaptic glutamate) is considered as a pulse train with a frequency of 5 Hz and amplitude of 0.2 mM. Choosing these values are based on experimental observations used by Tewari and Majumdar’s computational models (Tewari & Majumdar, 2012b; Tewari & Majumdar, 2012a).

**Figure 2.**
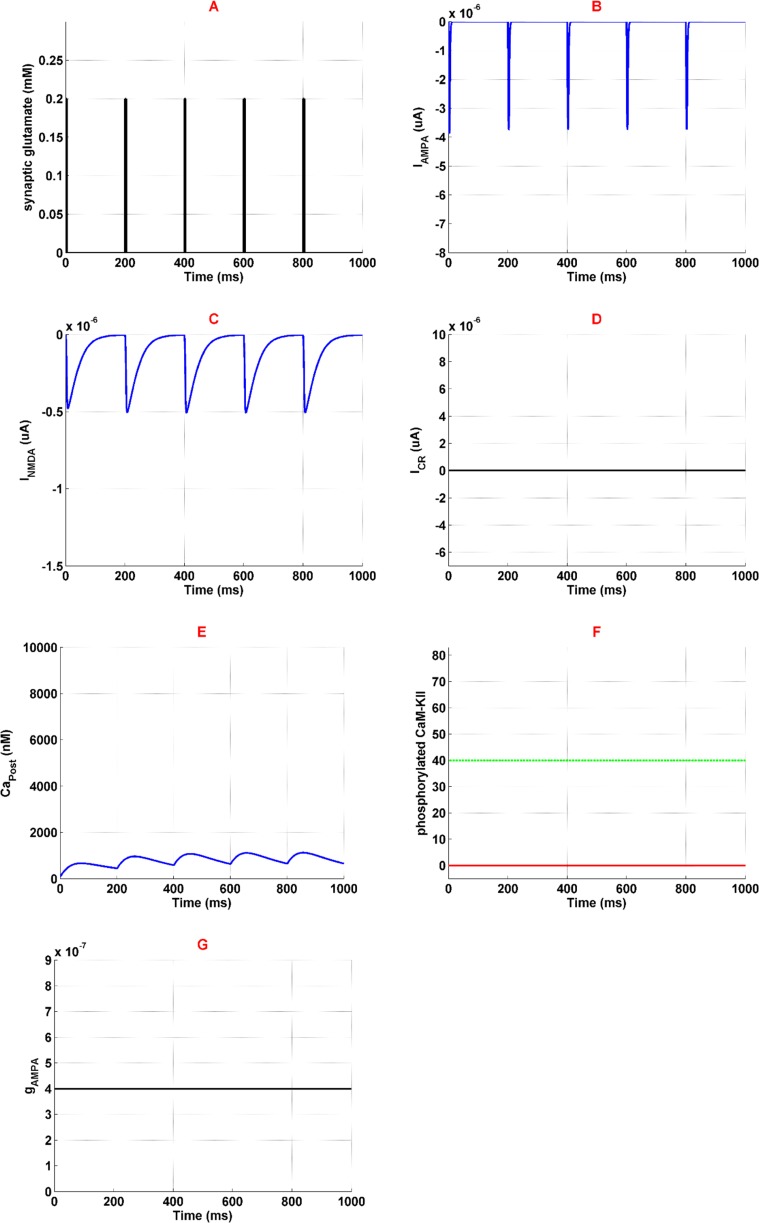
Activation of the neural components in normal condition Panel A shows stimulation signal which is synaptic glutamate. Panel B and panel C demonstrate AMPAR and NMDAR mediated currents. Panel D is the current mediated by VGCC. Panel E shows calcium concentration variation in the neuron. Panel F shows phosphorylated CaMKII in redline, and green line is the threshold beyond which AMPAR can phosphorylate. Panel G demonstrates AMPAR's conductance which is constant. Simulation time is 1 second in all panels.

Applying synaptic glutamate (Figure 2a) leads to AMPARs activation (Figure 2b) which depolarize membrane potential. Depolarized membrane allows NMDARs activation by removing Mg^2+^ blocking (Figure 2c). Since high-voltage calcium channels are considered in the model, calcium channels activation is 0 which is described in Figure 2d. Ions of Ca^2+^ can enter into the neuron through AMPARs and NMDARs activation. So, Ca^2^ concentration enhances in the neuron which is shown in Figure 2e. Entered Ca^2+^ can engage in CaMKII phosphorylation process, however, due to lack of enough calcium, this process is not observed here (Figure 2f). Based on simulation results, none of the AMPARs can phosphorylate (Figure 2g). Generally, this simulation can describe the normal condition which there is not any opioid’s effect in the synapse. In this simulation, LTP does not occur in the synapse due to lack of phosphorylated CaMKII and AMPARs.

**Figure 3.**
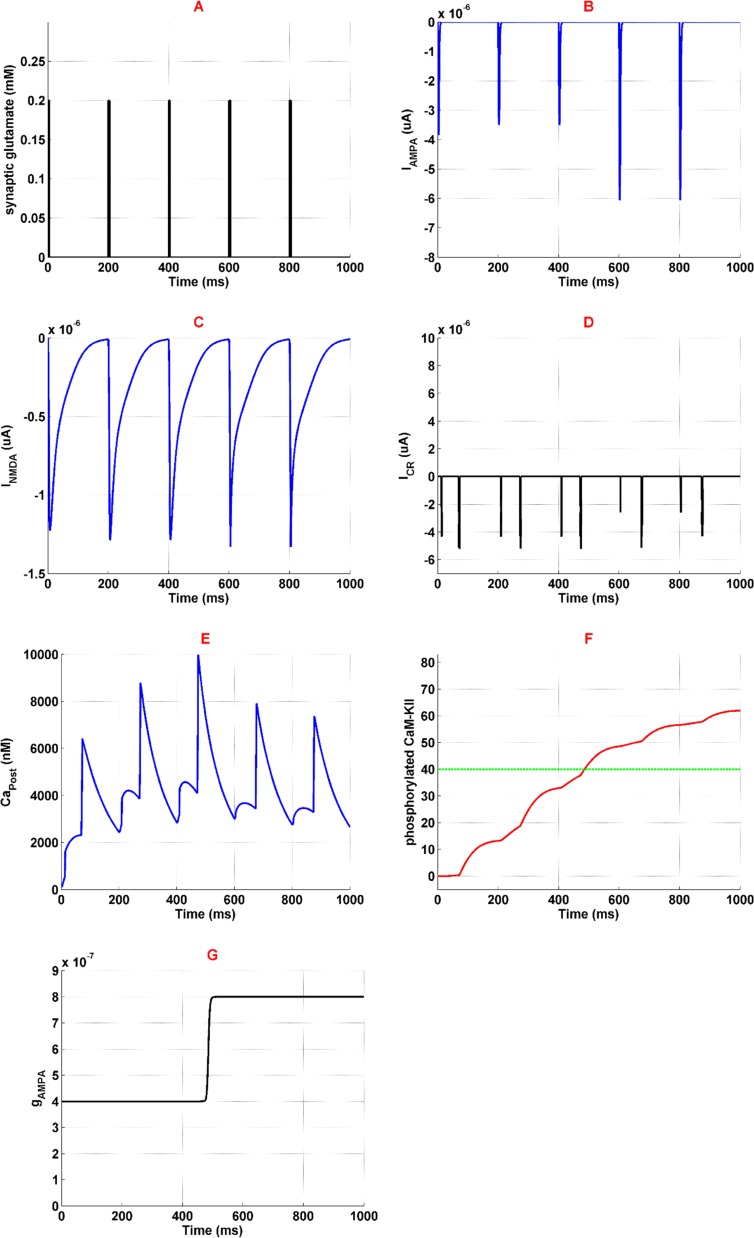
Activation of different neural components in the presence of 1 μM opioid Panel A shows glutamate concentration which is similar to the normal condition. Panel B is the current mediated by AMPAR. Panel C shows current mediated by NMDAR. Panel D is the VGCC's current. Panel F shows phosphorylated CaMKII in red-line and threshold for AMPAR phosphorylation in green line. Finally, panel G is the AMPAR conductance. Simulation time is 1 second for all panels.

Applying 1 *μM* of opioid increases NMDARs activation through inhibition of Mg^2+^ blocking and enhancement of receptors conductance. NMDAR mediated current enhances in this situation (Figure 3c). Thus, more calcium enters into the neuron (Figure 3e) which triggers CaMKII phosphorylation process (Figure 3f). Furthermore, more activation of NMDARs results in VGCCs activation. Figure 3d shows calcium current mediated by VGCCs. Exceeding phosphorylated CaMKII from a predetermined threshold can phosphorylate AMPARs. In fact, AMPAR conductance increases which is shown in Figure 3g. Thus more current is mediated by AMPARs (Figure 3b) that can work as a positive feedback for enhancement of calcium concentration in the neuron.

### 3.2. Comparing different factors on pathological LTP induction

Simulation results show that injection of opioid can induce LTP when normal LTP does not occur. Now we want to assess which components of the model are responsible in inducing such a pathological LTP. Based on these simulations we can suggest a way to prevent this type of pathological LTP.

Further simulations were performed to analyze different parameters involved in inducing pathological LTP. Normalized value for phosphorylated CaMKII and AMPARs are calculated in each simulation. The values are calculated by using the following Equation:

(17) $$Y_{Normalized}=\frac{Y-Y_{\text{Min}}}{Y_{Max}-Y_{Min}}$$

Where Y*_Normalized_* refers to the normalized value, *Y* is the parameter value, also *Y_Max_* and *Y_Min_* denote maximum and minimum values of the parameter, respectively. This normalization approach results in normalized values within [0–1] interval.

**Figure 4.**
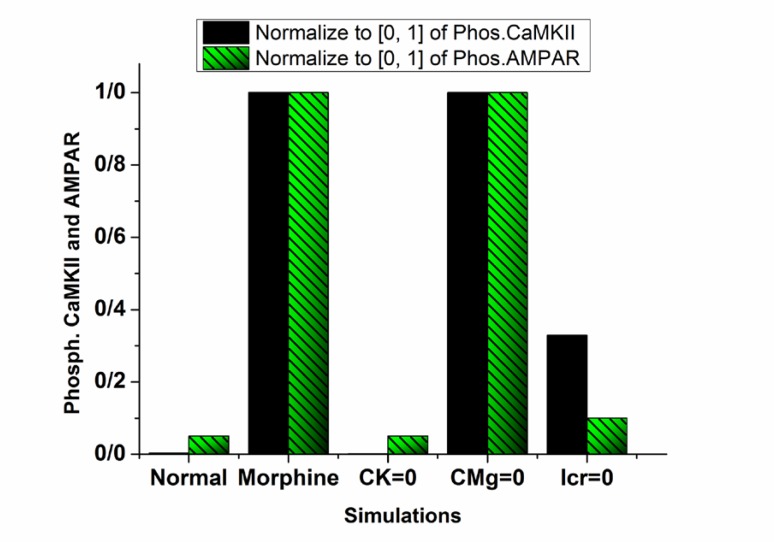
Normalized values for phosphorylated CaMKII and phosphorylated AMPAR in five different simulations The plain black bar is the phosphorylated CaMKII, and the green bar with black patterns is the phosphorylated AMPAR. Horizontal axes with “normal” label shows simulation in normal condition, “Opioid” label shows opioid injected condition, “CK=0” stands for the simulation in which opioid effect on NMDAR's conductance is assumed as 0, “CMg=0” describes the state that opioid effect on Mg^2+^ blocking is assumed as 0 and “Icr=0” refers to the case that current mediated by VGCC is assumed as 0.

Opioid-induced LTP were analyzed with 3 different assumptions: 1. Opioid does not modify NMDARs conductance; 2. Opioid does not have any infulence on Mg^2+^ blocking; and 3. VGCCs mediated current is 0. Simulation results according to the assumptions are shown in Figure 4. In normal condition, phosphorylated CaMKII and AMPAR is 0 which specifies there is not any step foot of LTP in the synapse. Presence of opioid leads to phosphorylation of CaMKII and AMPAR, and normalized values are in the high state. This simulation demonstrates the presence of LTP in the synapse.

It can be seen that LTP does not occur in the synapse by neglecting opioid effect on NMDAR’s conductance (CK=0 label in Figure 4). Although, neglecting opioid’s effect on Mg^2+^ blocking (CMg=0 label in Figure 4) is not a critical parameter, knocking out VGCC’s activation leads to attenuation of the LTP (Icr=0 label in Figure 4). These results show that enhancement of NMDAR’s conductance besides VGCC’s activation is a fundamental parameter in inducing pathological LTP. Therefore in the next part, constants for VGCC’s activation and NMDAR’s conductance are changed for further simulations.

**Figure 5.**
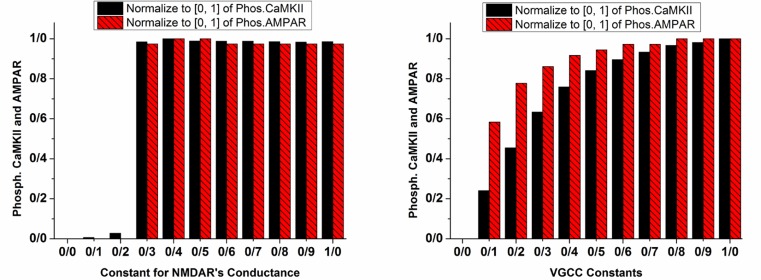
Phosphorylated CaMKII and AMPAR with variable constants for NMDAR's conductance (left panel) and VGCC (right panel) Normalized values for phosphorylated CaMKII are in plain black bars, and normalized values of phosphorylated AMPAR are in patterned red bars. Left panel demonstrates the conditions in which NMDAR's conductance coefficient varies with 0.1 increments. The right panel shows the conditions in which current mediated by VGCC is multiplied by the variable constant with 0.1 increments.

The left panel of Figure 5 shows the normalized value for phosphorylated CaMKII and AMPAR with a variable coefficient of NMDAR’s conductance. It can be seen that when the coefficient is 0.2, there is not any phosphorylation; however, when it is 0.3, CaMKII and AMPAR phosphorylate. It means that attenuation of LTP by manipulating NMDAR’s conductance is impossible. We can whether completely block LTP or not, and there is nothing in between. However, according to the simulation results shown in right panel of Figure 5, increment in VGCC mediated current strengthens LTP. As a matter of fact, increasing channels activation constant intensifies LTP.

**Figure 6.**
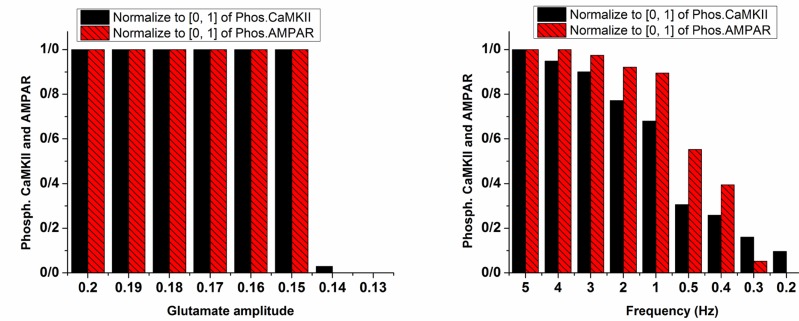
Phosphorylated CaMKII and AMPAR with variable amplitude (left panel) and frequency of glutamate (Right panel) Normalized values for phosphorylated CaMKII are in plain black bars, and normalized values of phosphorylated AMPARs are in patterned red bars. In the left panel, glutamate amplitude decreased from 0.2 to 0.13 mM. In the right panel, frequency of glutamate pulse train decreased from 5 to 0.1 Hz.

Besides opioid’s direct effect on postsynaptic neurons, change in frequency and amplitude of stimulation signal which are considered as synaptic glutamate can alter pathological LTP. The left panel of Figure 6 shows the variation of glutamate on the CaMKII and AMPAR phosphorylation process. This simulation shows that reducing glutamate amplitude by 0.01 increments from 0.2 does not lead to inhibition of LTP until glutamate amplitude reaches 0.14. At this value, phosphorylated CaMKII and AMPARs decline rapidly, and LTP does not occur. However, a gradual decrease in stimulation frequency leads to gradual descend in LTP induction shown in the right panel of Figure 6.

## 4. Discussion

Our simulation results support the hypothesis that opioids consumption can lead to pathological memory formation in hippocampal neurons through induction of LTP. To explain this issue, we developed a computational model in which CaMKII and AMPAR phosphorylation processes are considered as foot prints of LTP induction.

Experimental observations (Chen & Huang, 1992; Chen & Marine, 1991; Kow et al., 2002; Mao, 1999) indicate the opioids effects on NMDARs. Enhancement of NMDAR mediated current is a byproduct of opioids presence in synapses. Our simulation results showed that enhancement of NMDAR’s conductance is more efficient in inducing LTP compared to inhibition of Mg^2+^ blocking through opioids. In other words, despite the inhibitory effect of Mg^2+^ blocking on NMDAR mediated current, we observed that the main reason of LTP induction is enhancement of NMDA receptors conductance. Furthermore, our results suggest that prevention of pathological LTP can be achieved by blocking NMDAR’s conductance. NMDAR’s conductance acts like a none-or-all switch in inducing LTP. Therefore, it is impossible to attenuate pathological LTP by decreasing NMDAR’s conductance. However, we may completely block it using this approach.

Bongianni et al. (1986) and Mishra et al. (2017), through their experimental observations, suggested that blocking VGCCs leads to preventing dependency on opioids. In this research, we obtained the same result but in a different way, i.e., we showed the role of VGCCs in forming pathological LTP. Our simulation results demonstrate that by inhibiting VGCCs, pathological LTP is attenuated. Also, we may infer that by decreasing VGCC activation, pathological LTP is gradually lessened. One may be able to apply this finding in cases where one intends or prefers to attenuate pathological LTP rather than blocking it.

On the other hand, disinhibitory effect of opioid increases glutamate concentration in the synaptic cleft. Therefore, it is reasonable to assume that changes in the amplitude and the frequency of the stimulation signal in the postsynaptic neuron are due to the presence of the opioid. Simulation results demonstrate that increasing the frequency and the amplitude of the stimulation signal results in LTP induction. There are different experimental observations that support these simulation results (Akaishi et al., 2000; Capogna et al., 1993; Caudle & Chavkin, 1990; Cohen et al., 1992; Kauer & Malenka, 2007; Rezai et al., 2013).

These observations indicate that opioid inhibits the GABAergic signal that leads to enhancement of glutamate concentration in the synapse. Furthermore, our simulation results suggest that reducing synaptic glutamate can prevent pathological LTP induction. Amplitude and frequency of glutamate release are parameters that will help in preventing or attenuating LTP. Since the source of glutamate is mainly from presynaptic neurons, inhibition of presynaptic pyramidal neurons may help prevent pathological LTP induction. On the other hand, one of the fundamental functions of the glial cells is reuptake of the synaptic glutamate. Therefore, we also suggest considering the role of glial cells in preventing pathological memory formation. This goal can be achieved through stimulating glutamate transporters. Using this approach, synaptic glutamate can be reduced, and this can result in reducing stimulation of the postsynaptic receptors.

In this paper, we introduced a computational model for postsynaptic neurons in CA1 region of the hippocampus to examine LTP induction due to opioids. The model is consisted of a spine which produces EPSPs. NMDAR, AMPAR and VGCC’s mediated currents are also considered in the model. Calcium ions flow into the neuron through these channels and receptors. Elevation of calcium concentration leads to CaMKII and AMPAR phosphorylation. We assumed that CaMKII and AMPAR phosphorylation are foot prints of LTP induction.

Simulation results of the presented model indicate that presence of opioids can lead to LTP induction in a situation that normal LTP does not occur. This finding supports the idea that opioids may form a memory that is directly linked to drug consumption. Therefore, one of the primary triggers of relapse in withdrawal time is this pathological memory. Furthermore, we observed that, by decreasing VGCC’s activation, NMDAR’s activation, and glutamate concentration in the synapse, pathological LTP reduces and it is even possible to prevent the pathological LTP.

## Ethical Considerations

### Compliance with ethical guidelines

Since the research was a simulation one, the authors did not need to get any ethical approvals.

## Appendix.

**Table:** Numerical values of the model parameters

**Variable**	**Description**	**Value (Units)**	**Source**
R_m_	Actual resistance of the spine-head	0.79*10^5^ MΩ	(Tsodyks & Markram, 1997)
V^rest^_post_	Postsynaptic resting membrane potential	−70 mv	(Tsodyks & Markram, 1997)
*τ*_post_	Postsynaptic membrane time constant	50 ms	(Tsodyks & Markram, 1997)
V_AMPA_	AMPAR reversal potential	0 mv	(Destexhe et al., 1998)
α_AMPA_	AMPAR forward rate constant	1.1 μMs^−1^	(Destexhe et al., 1998)
β_AMPA_	AMPAR backward rate constant	190s^−1^	(Destexhe et al., 1998)
V_NMDA_	NMDAR reversal potential	0 mv	(Destexhe et al., 1998)
F	Faraday’s constant	96487 Cmol^−1^	(Moradi et al., 2013)
R	Real gas constant	8.314 J/K	(Moradi et al., 2013)
T	Absolute temperature	293.15 K	(Moradi et al., 2013)
Z	Valence of Mg^2+^	2	(Moradi et al., 2013)
δ	Relative electrical distance of the binding site of Mg^2+^ from the outside of the membrane	0.8	(Moradi et al., 2013)
K_0_	IC_50_ at 0 mV	4.1 μM	(Moradi et al., 2013)
[Mg^2+^]_0_	Concentration of Mg^2+^ n the extracellular fluid	1 μM	(Moradi et al., 2013)
α_NMDA_	NMDAR forward rate constant	7.2 *10^4^ M^−1^ s^−1^	(Destexhe et al., 1998)
β_NMDA_	NMDAR backward rate constant	6.6 s^−1^	(Destexhe et al., 1998)
K	Steepness of speed of voltage-dependent gating of channel	0.007	(Moradi et al., 2013)
V_0_	The V_m_ at which g_VD,∞_ is equal to 0	−100 mv	(Moradi et al., 2013)
τ_g_	Time constant which determines the speed of voltage-dependent gating of channel	0.05 ms	(Moradi et al., 2013)
